# Hospitals and State Aid.—III

**Published:** 1911-07-01

**Authors:** 


### HOSPITALS AND STATE INSURANCE.

### HOSPITALS AND STATE AID.—III.

CONDITIONS UNDER WHICH GOVERNMENT AID IS GIVEN TO HOSPITALS.

(By our Special Commissioner.)

### SOME CROWN COLONIES.

The hospital system that prevails in most of our Colonies which have not yet received the rights of self-government, differs in some respects from that which obtains in the Dominions. In the Crown Colonies the greater number of the hospitals are Government institutions which depend for their upkeep solely upon the large grants which are made to them by the Department of Health out of the general revenue of the colony. In many cases they receive a part of their revenue from payments made by patients, but the scale on which such patients' charges have been calculated is generally so low that the receipts from this source are small. As Government institutions, these hospitals are controlled solely by Government officials, and the public has no voice whatever in their management. At their head is usually a medical man appointed by the Governor upon the recommendation of the medical officer of health; this superintendent is well paid, and in the smaller colonies, where the number of medical men is necessarily limited, he is permitted to do private practice. The nurses and lay staff are under his control; his colleagues on the medical staff are appointed and removed by the head of the department.

In other cases—as for example in the Bahamas—there is a special board appointed by the Governor in Council to which is delegated the duty of administering the hospitals. In the self-governing colony which we have included in this article (Barbados) the hospital is to all intents and purposes a Government institution, although it is controlled by a special board. In certain Crown Colonies, such as Gibraltar and Malta * and the smaller islands, the hospitals are frankly departmental, being usually either naval or military. With these we have not thought it necessary to deal.

#### BRITISH GUIANA.

This colony possesses some admirable hospitals and charitable institutions. The public hospital at Georgetown, with 536 beds, is mainly supported out of the general revenue, the income from all sources being $90,584, of which Government contributed $81,663, the difference being made up by patients' fees. The public hospitals at Suddie, Bartica, New Amsterdam, and Morawhanna, the Leper Hospital at Mahaica, and a Seamen's Hospital at Georgetown are all similarly supported. The control of such institutions is vested in officials paid by the Department of Health. During 1908-09 a sum of £41,535 8s. 2d. was granted to the hospitals. There are a large number of estates' hospitals, of the cottage type, which, although some of them are purely private, are under the supervision of the Surgeon-General.

#### FIJI.

There is a fine Government Colonial Hospital at Suva with 130 beds, which is wholly a State supported institution, and has a medical school for the training of native diplomates attached to it. Patients are charged moderate fees. An interesting account of this institution, and of the provincial hospitals, will be found in Dr. St. Johnston's paper, "Fiji: Its Hospitals and Diseases" (The Hospital, vol. xliv., pp. 23, 75, 1908).

#### JAMAICA.

A public medical service was organised in 1868, when fifteen salaried district medical practitioners were appointed. At present there are thirty-nine medical districts and thirty-eight district officers, one district being vacant, as there are no more practitioners available. The district officer must take charge of the pauper sick and of the hospital if there is one in his district, and is allowed private practice. At the public hospital there is a superintending medical officer who is assisted by a senior medical officer, two resident medical officers, and a supernumerary. The receipts of the whole department in 1910 were £1,131 odd, and the expenditure was £57,843 odd. There is a ticket system in force by which provision is made for the treatment of those who, while not paupers, cannot afford the lowest fee charged for private attendance (4s.). The following hospital regulations are drawn up for public general hospitals which are supported out of the general revenue:—

Patients who are on the Parochial Pauper Roll shall be admitted free, provided the medical officer considers them fit subjects for hospital treatment.

Other applicants, not on the Parochial Pauper Roll, but who are unable to contribute towards their maintenance, shall be admitted into hospital free of all charges on the written recommendation of the Custos or Chairman of the Parochial Boards or the Inspector of Poor or Clerk of Parochial Board or of any member of the Board of Official Visitors of the hospital, provided on examination they are found fit subjects for admission and there is accommodation available. Creole and coolie labourers shall be admitted free of charge.

The ticket system has been found to work well. By Government proclamation in 1880 it was provided that the Governor will, on the nomination of the Chairman of a Parochial Board or otherwise, appoint gentlemen to be distributors of medical relief tickets, who will be furnished by the superintending medical officer with tickets of the respective values of 3s. and 2s. as respects all parishes except Kingston, and of the values of 2s. and 1s. as respects Kingston. Persons unable to pay the minimum fee of 4s. may, if considered deserving of relief, obtain from these gentlemen one of these tickets which on presentation to the medical officer at the Government dispensary will entitle him on payment of the fee represented on the ticket to medical advice and medicine. A separate ticket must be presented on each occasion; in case the patient requests home attendance he must pay mileage in addition to the ticket fees at the rate of 1s. a mile going and 6d. a mile returning. In the case of prescriptions prepared at Government dispensaries or with Government drugs, one-third of the fee received goes to the

* At Malta there is a Civil Hospital with 200 beds, which is under the Government Charitable Institutions Department, and which receives paying patients; there is also the Santo Spirito Hospital (72 beds), a similar institution, and the Merchant Seamen's Hospital at Floriana (25), also a Government Hospital. At Gibraltar there is a very excellent Civil Hospital, of which particulars are given in Burdett's "Hospitals and Asylums of the World."

Government and the remaining two-thirds to the doctor. Out-patients at a public hospital must bring a signed recommendation from some responsible person to the effect that they are proper persons for out-patient relief. In-patients must contribute towards the costs of treatment in certain cases. The hospitals are (with one or two exceptions) wholly supported by grants from the general revenue; all this expenditure is classed under the general head of medical, which last year totalled to nearly £60,000. The public general hospitals in the island are as follows: Morant Bay (25), Hordley (25), Port Antonio (155), Buff Bay (40), Annotto Bay (230), Port Maria (100), St. Anns Bay (20), Falmouth (20), Lucea (18), Savanna-la-Mar (10), Black River (25), Mandeville (25), Chapelton (30), Montego Bay (25), Linstead (6), Lionel Town (110), Spanish Town (70), Cave Valley (6).* The large general hospital at Kingston has a special Board of Visitors, and has attached to it a honorary staff. The resident staff is paid by the Government, and it is for all practical purposes a Government institution, though it has benefited largely by private donations, such as the Rietti bequest, which has enabled a new operating-room and Rietti ward to be built. The Victoria Jubilee Lying-in Hospital at Kingston was founded as a voluntary hospital, but the Legislature supplemented the building fund by a considerable grant, and now maintains it by annual vote. It accommodates eighteen patients, who must either be too poor to pay or must pay at the rate of 15s. for ten days and 1s. for every day after that period. There is a public dispensary which is supported by a society. Special regulations regarding the extirpation of mosquitoes and vermin have been drawn up for the hospitals. All hospitals must keep "one or more cats." The Jamaican hospitals serve the Turks and Caicos Islands as well.

### BERMUDA.

There is a cottage hospital at Pembroke which is not in any way endowed, but entirely dependent on voluntary contributions, patients' fees, and Hospital Sunday Fund contributions. The governor and an elected board of management control the institution. The hospital was opened in 1894, at a cost of about £1,500, and is now well equipped. Patients are charged £1 11s. per week in the wards. There is a Government supported and controlled infirmary asylum.

### GRENADA.

The hospitals are Government institutions, managed by Government officials, and supported partly out of the general revenue and partly by receipts from patients. There are two district hospitals, one at Carriacou, with twenty-eight beds, and one at St. Andrew, near Grenville. There is a Yaws Hospital at St. George's, to which any person afflicted with the disease can be summarily removed at the request of a medical officer, justice of the peace, constable, or minister of religion. The main hospital (known as the Colony Hospital) is at St. George, and was thoroughly reconstructed in 1897. Cases are admitted on the written recommendation of a member of the Council, a medical man, justice of the peace, or clergyman, and if paupers are treated gratuitously. In other cases they are charged fees at the rate of 1s. per day in the ordinary, and 3s. to 5s. per day in the private ward. A guarantee of expenses must in each case be given before the patient is admitted. At the hospital dispensaries prescriptions are made up for the general public at fixed rates.

* The figures in brackets denote the number of beds.

### TURKS AND CAICOS ISLANDS (see JAMAICA).

### TRINIDAD AND TOBAGO.

The Colonial Hospital (320 beds) in Trinidad is a Government institution, taking in patients who are unable to pay for private medical attendance. The charge for such patients is 1s. per day; merchant seamen and private patients are charged from 2s. to 5s. per day. The Government grant in 1909 exceeded £10,000. The Leper Asylum is maintained by Government. A Tuberculosis Dispensary was established in 1905: it is entirely a voluntary institution. At Arima are two hospitals, one for yaws, the other a general cottage hospital: at St. Fernando is another hospital with 130 beds, all three are Government institutions. Tobago has a Colonial Hospital at Scarborough. The staffs of these institutions are particularly well paid.

### MAURITIUS.

The hospitals are Government institutions. There is a general board of health on which several lay members sit who are appointed by the municipal corporation, the Chamber of Agriculture, and the Chamber of Commerce, while one is nominated by the medical men of the island. There is a central office des Institutions Charitables, and there are several religious associations which do nursing work. The Civil Hospital at Port Louis (250 beds) is under the Director of the Health Department, and special terms are arranged for Government patients.

### ST. VINCENT.

There is a Colonial Hospital and a Pauper Asylum: the former is at Victoria Park, and is a general public hospital, to which is attached a Government dispensary. One of the hospital blocks was built and is maintained out of a special fund bequeathed by the late James Graham, of Kingstown. It contains four small wards for better-class patients, who are charged from 4s. 6d. per day. In the other wards the charges vary from 1s., pauper patients being admitted free. The institution, with the exception of the Graham Ward, is supported and controlled by Government. In 1903-04 the expenditure on hospitals and other charities was £4,300; in 1907-08 it was only £3,200.

### BAHAMAS.

By special local Act a Poorhouse Hospital was established in Nassau in 1809 for the reception of poor patients from the Island of New Providence. By Act of 1845 it was placed under a special board of three commissioners annually appointed. Later on the number was raised to seven. These commissioners have not only to manage the hospital, but have to administer "all affairs relative to the support of the aged and infirm poor on the several out-islands." The hospital has a public dispensary attached to it, and is an infirmary for poor patients as well as a hospital. In 1887 the new Victoria Ward was built, and in 1893 a section of the Bahamas General Hospital was re-christened "The Victoria Jubilee Hospital." In 1897 a fine new mental hospital was built; in 1902 the Alexandra Infirmary was established. The hospital is supported by Government grant of £6,000, but the control of the institution is largely in the hands of the directors, who are appointed under special Act.

### BARBADOS.

There is a General Hospital Board of which the Governor is President and the Bishop Deputy Chairman, while the President of the Council, the Attorney-General, the Solicitor-General, and the Speaker of the Assembly are ex-officio members: four members appointed by the Legislative Council, and six by the Assembly, while the rest of the board consists of Life Directors elected by the subscribers. The General Hospital is at Bridgetown, and was established in 1840 by private subscription, but is an endowed institution with 227 beds, which is liberally supported by the Legislature. The management of the hospital is wholly in the hands of the Directors, who appoint the staff. Natives pay 3s. to 7s. 6d. per day; foreigners £1 per day. There is a public lazaretto, which is supported by the Legislature, and there are several flourishing charitable associations, which are dependent on voluntary contributions. No hospital statistics are available.

### THE CAYMAN ISLANDS.

A very much underpaid medical department exists. There is a Government dispensary at Georgetown, but no hospital.

### WEST AFRICAN COLONIES.

Hospitals are maintained wholly by Government, though a few small mission hospitals exist. There are two good hospitals in Lagos besides several dispensaries. Sierra Leone possesses a good general hospital in Freetown, a nursing home in Freetown, and special hospitals at Kissy and Bonthe Bay. There are several hospitals, or rather hospital dispensaries, in each of the Protectorate districts, all supported by Government. The Princess Christian Cottage Hospital is supported by voluntary subscriptions (£1,234 in 1901), and the management is by a local committee. The Nursing Home is also managed by a local committee consisting of three Government representatives and three chosen by the subscribers, but a grant is made towards the funds by the Government.

### ST. LUCIA.

The Victoria Hospital, opened in 1887, is close to Castries, and has a capacity of 150 beds. The Colonial Surgeon is in charge, and the cost of erection (£8,000) was defrayed by Government. "Admission of patients is restricted to cases requiring surgical assistance, acute diseases requiring constant attention, and chronic diseases requiring special treatment. Paying patients are admitted at the written request of any Castries householder, provided there is accommodation available, or any master of a vessel lying in the harbour. The charge varies from 2s. to 4s. per diem. Under Section 42 of Ordinance No. 289 of 1871, a ward in this hospital is set aside for the reception of sick seamen. The expenses of this ward are kept separate from the hospital expenditure, and are defrayed by a special hospital levy of one penny per ton on all vessels anchoring in the port of Castries. There are Government supported and controlled hospitals on Rat Island (Yaws Hospital), at Soufrière (twenty beds), Vieux-Fort (thirty-four beds), and Dennery (thirty-five beds), besides several Government dispensaries. There is a special Board of Health, whose regulations are subject to the approval of the Governor in Council. The Chairman of the town board has a seat on this board, the members of which are appointed by the Governor at discretion. This board receives a yearly grant from the Government revenues. The hospitals are under the medical department, at the head of which is the Colonial Surgeon, who is a member of the Executive Council with the title of honourable. All hospital medical officers are allowed private practice and fees at inquests. The medical and charitable expenditure of the colony was £6,993 in 1897, and £7.024 in 1902. No statistics showing hospital revenue apart from Government grants are available.

### EAST AFRICA AND UGANDA.

The hospital and medical service was reorganised in 1903, when the Uganda Railway Medical Service was taken over by the Government. There are hospitals supported and controlled by Government at Mombasa, Entebbe and Nairobi, and native hospitals and dispensaries at every station. At the hospitals Government officials with a salary of less than £250 per annum are charged 2½ rs. per diem; over that income but below £400, 3 rs. per diem; and over £400 from 4 rs. upwards. Patients who are not officials are charged 12 rs. per day for a bed in a single, 7 rs. per day for one in a double, and 5 rs. per day for a bed in an ordinary ward. All patients, whether official or not, have to pay for medical comforts. There is a voluntary nursing association. At Mzizmia is a hospital supported by a mission, another at Kabarole, and another at Mengo.

### CENTRAL AFRICA.

The Government Civil Hospital at Blantyre, Nyasaland, is a small cottage type with six beds. The Government subsidy is £10 per bed per year. It was rebuilt at Government expense three years ago. At Fort Johnston is a two-bedded Government hospital, which receives an annual grant of £17. At Zomba there is a civil hospital and a mission maternity hospital, both voluntary institutions with a special board. The Zomba Civil Hospital receives a Government grant of £50 per year. All these institutions charge patients who are admitted, the charge being at the rate of 10s. per day.

### HONGKONG.

There are three voluntary hospitals supported by missions. The Civil Hospital (150 beds), the Kennedy Town Hospital (50), the Maternity (10), and the Victoria Hospital for Children (41 beds) are Government institutions, wholly State supported and managed. Patients are charged at the rate of from half a dollar to eight dollars per day, unless they are absolute destitute.

### BRITISH HONDURAS.

Three small district hospitals, one at Orange Walk (16 beds), one at Punta Gorda, and a third at Corozal (12), all Government institutions. The public hospital at Belize (50 beds) was originally a seamen's, but is now also a Government hospital. Patients are charged from sixpence (nominal fee) to 8s. per day.

### LEEWARD ISLANDS.

Eight hospitals exist: St. Kitts (134), Nevis Infirmary (43), Dominica (70), Yaws Hospital (20), Chateau (51), Montserrat (12), Antigua (132), and Virgin Islands (20). They are controlled by boards of management locally elected, on which sit Government nominees. A Government grant is made to each institution, and all the hospitals are subject to annual inspection by the Government medical officers; otherwise the management is left entirely in the hands of the local boards.

### MALAY STATES.

The hospitals are entirely State supported, all receiving paying patients as well as poor. There are a few mission hospitals, which are kept up by voluntary grants made by the parent societies.

